# Alterations of oral microbiome and metabolic signatures and their interaction in oral lichen planus

**DOI:** 10.1080/20002297.2024.2422164

**Published:** 2024-10-30

**Authors:** Li Yan, Jingyi Xu, Fangzhi Lou, Yunmei Dong, Shiping Lv, Ning Kang, Zhuoyan Luo, Yiyun Liu, Juncai Pu, Xiaogang Zhong, Ping Ji, Peng Xie, Xin Jin

**Affiliations:** a College of Medical Informatics, Chongqing Medical University, Chongqing, China; b Chongqing Key Laboratory of Oral Diseases and Biomedical Sciences, College of Stomatology, Chongqing Medical University, Chongqing, China; c College of Stomatology, Chongqing Medical University, Chongqing, China; d NHC Key Laboratory of Diagnosis and Treatment on Brain Functional Diseases, The First Affiliated Hospital of Chongqing Medical University, Chongqing, China

**Keywords:** Oral lichen planus, oral microbes, metabolites, multi-omics data, machine learning

## Abstract

**Background:**

Oral lichen planus (OLP) is a chronic oral mucosal inflammatory disease with a risk of becoming malignant. Emerging evidence suggests that microbial imbalance plays an important role in the development of OLP. However, the association between the oral microbiota and the metabolic features in OLP is still unclear.

**Methods:**

We conducted 16S rRNA sequencing and metabolomics profiling on 95 OLP patients and 105 healthy controls (HC).To study oral microbes and metabolic changes in OLP, we applied differential analysis, Spearman correlation analysis and four machine learning algoeithms

**Results:**

The alpha and beta diversity both differed between OLP and HC. After adjustment for gender and age, we found an increase in the relative abundance of *Pseudomonas*, *Aggregatibacter*, *Campylobacter*, and *Lautropia* in OLP, while 18 genera decreased in OLP. A total of 153 saliva metabolites distinguishing OLP from HC were identified. Notably, correlations were found between *Oribacterium*, specific lipid and amino acid metabolites, and OLP’s clinical phenotype. Additionally, the combination of *Pseudomonas*, *Rhodococcus* and (±)10-HDoHE effectively distinguished OLP from HC.

**Conclusions:**

Based on multi-omics data, this study provides comprehensive evidence of a novel interplay between oral microbiome and metabolome in OLP pathogenesis using the oral microbiota and metabolites of OLP patients.

## Introduction

Oral lichen planus (OLP) is a prevalent chronic inflammatory disease affecting the mucosa of the mouth [1], frequently observed in women aged 30 to 60, with a prevalence ranging from 0.49% to 1.43% [2,3]. Lesions typically feature bilateral whitish striae, occasionally with erythema or erosions. The disease progresses slowly and persistently, often leading to recurrent pain and impairment of oral functions, which significantly reduces the quality of life for patients [4]. Moreover, OLP carries a potential risk of malignant transformation, categorizing it as oral potentially malignant disorders (OPMDs). Recent studies have indicated a malignant transformation rate of about 1.14% [5]. Although several hypotheses have attempted to explain its pathophysiological mechanisms, the molecular basis of OLP remains largely unclear [6]. Identifying specific molecular markers for OLP is critical, given the lack of objective evidence for screening and treatment [7], enabling more accurate diagnoses and targeted therapies.

The microbiota could maintain oral homeostasis by regulating the immune response [8]. There is considerable evidence that immune dysregulation plays a role in the pathogenesis of OLP, despite the fact that its exact cause is still not fully understood [9]. Viruses, fungi, and various bacteria create an unstable microenvironment inside the mouth, an organ directly exposed to external pathogens [10]. Disturbances in the oral microbiome composition may lead to dysregulation of the patient’s immune system, resulting in OLP [11,12]. Research indicates that OLP and HC groups showed similar community richness and diversity [13]. Yu et al. reported that *Lautropia* and *Gemella* were more enriched in erosive OLP, whereas *Haemophilus* and *Neisseria* were more enriched in non-erosive OLP, with both *Abiotrophia* and *Oribacterium* being enriched across all OLP types [14]. A positive correlation was also found between the relative abundance of *Streptococcus*, *Haemophilus*, *Rothia*, and *Granulicatella* in saliva and the severity of OLP [15].

Salivary metabolites play a key role in maintaining the balance between various oral microorganisms and their human hosts. The secretion of bacterial metabolites can directly affect the health status of the oral cavity and the nature of the immune interface in the oral microecosystem [16]. Thus, salivary metabolomics offers insights into host–microbe interactions and provides information about metabolic characteristics associated with disease [17]. Research has confirmed the association of changes in the oral microbiome or its metabolism with numerous immune and inflammation-related diseases, such as autoimmune disease, inflammatory bowel disease and rheumatoid arthritis [18–21]. A previous study has identified differences in saliva metabolic profile in OLP. Wang et al. discovered 19 metabolites differing between OLP and HC, notably in amino acids, carnitines, and lipids. Furthermore, they found that glycerophospholipid metabolism, arginine and proline metabolism, alanine, aspartate, and glutamate metabolism, D-glutamine and D-glutamate metabolism, and taurine and hypotaurine metabolism are closely associated with OLP pathogenesis [22].

In contrast, previous studies have mainly examined the metabolomics of urine and blood, or the composition of the microbiota, while the crosstalk between oral microbes and host metabolites remains largely unknown in the pathogenesis of OLP. To address these limitations, an OLP cohort and healthy controls were analyzed for 16S rRNA gene amplicon sequencing and untargeted liquid chromatography-tandem mass spectrometry (LC-MS) metabolomic profiling of saliva samples. By integrating microbiome and metabolome data, the distinct microbial and metabolic signatures associated with OLP were charactrized, enhancing our understanding of how microbial communities affect the host metabolic state [23,24]. In addition, a novel combinatorial marker panel that could distinguish OLP from HC was identified, which is of possible etiological and diagnostic importance.

## Materials and methods

### Participants and sample collection

In total, 200 saliva samples were collected from 95 OLP patients and 105 healthy controls between February 2021 and August 2022 at the Stomatological Hospital of Chongqing Medical University. All patients provided written informed consent prior to participation in the study. This study was approved by the Affiliated Hospital of Stomatology, Chongqing Medical University (GCP) (Ethics NO. 2021–4). Patients who satisfied the following criteria were enrolled in the OLP group: (1) age between 18 and 80 years. (2) meeting the diagnostic criteria in the OLP diagnosis [25]. (3) voluntarily participated in this study with informed consent and demonstrated good compliance. Exclusion criteria included: (1) accompanied by other oral mucosal disorders; (2) accompanied by systemic severe acute infections; (3) systemic diseases (e.g. diabetes mellitus, cardiovascular diseases) with long-term regular medication to control the condition; (4) women during pregnancy and lactation; (5) those who have used antibiotics or glucocorticoids in the last 3 months. The inclusion criteria for healthy controls were as follows: (1) individuals found to be healthy upon physical examination, matched in age and sex with the OLP cases; (2) no obvious oral disorders or systemic diseases.

Saliva samples were collected under unstimulated conditions during the morning hours between 8:00 and 11:00. Each participant provided 5 mL of saliva in sterile conical tubes free of DNase and RNase. Participants were instructed not to eat or drink anything for at least 3 h before sampling. For testing oral microbials and metabolites, the collected saliva samples were divided into two parts. The samples were immediately kept on ice and transported to the laboratory. Upon arrival at the laboratory, 300 μL of the supernatant was transferred into Eppendorf tubes and stored at −80°C until further analysis.

### Measurements of clinical parameters

During the visit, trained staff collected the patients’ demographic and clinical information. The severity of oral lichen planus was assessed using the RHU scale, which assigns weighted scores to three types of lesions: reticulation, hyperemia/erythema, and erosion/ulceration, with the total score reflecting the combined assessment. Reticular lesions are scored from 0 to 2, with 0 indicating no reticulation, 1 for reticulation covering less than 50% of the mucosal surface, and 2 for reticulation encompassing 50% or more. Erythema/hyperemia and erosion/ulceration are quantified in square centimeters to detect subtle changes in lesion area sensitively. The formula is as below: The severity score for OLP = R (total score for reticulated lesions) * 1 + h (total score for hyperemia lesions) * 1.5 + U (total score for ulceration lesions) * 2. Additionally, the RHU scale has been proven to be a valid and reliable method for severity measurement in patients with OLP [26].

### DNA extraction, 16S rRNA gene amplicon sequencing and data processing

Total microbial genomic DNA was extracted from whole saliva samples using the FastDNA® Spin Kit for Soil (MP Biomedicals, Norcross, US) following the provided guidelines. The DNA’s quality and concentration were assessed using 1.0% agarose gel electrophoresis and a NanoDrop® ND-2000 spectrophotometer (Thermo Scientific Inc., USA), and samples were stored at −80°C for subsequent analysis. For amplification of the V3-V4 regions of the bacterial 16S rRNA gene, primer pairs 338F (5’338F (5”-GGACTACHVGGGTWTCTAAT-3’) [27] were utilized in an ABI GeneAmp® 9700 PCR thermocycler (ABI, CA, USA). The components of the PCR reaction mixture are 4 μL of 5× Fast Pfu buffer, 2 μL of 2.5 mm dNTPs, 0.8 μL of each 5 μL primer, and 0.4 μL of Fast Pfu polymerase. Additionally, 10 ng of template DNA is included, with ddH_2_O added to achieve a total volume of 20 μL. The PCR amplification cycling conditions included initial denaturation at 95°C for 3 min, 27 cycles of denaturation at 95°C for 30 s, annealing at 55°C for 30 s, and extension at 72°C for 45 s, as well as single extension at 72°C for 10 min, and end at 4°C. Triplicate amplifications were performed on all samples. The PCR products were purified from a 2% agarose gel, quantified using a Quantus™ Fluorometer (Promega, USA), and prepared for sequencing. Equal molar concentrations of purified amplicons were sequenced on an Illumina PE300 platform (Illumina, San Diego, USA) by Majorbio Bio-Pharm Technology Co. Ltd. (Shanghai, China), following standard protocols. FastP (0.19.6) [28] was used to quality filter the sequences after demultiplexing, and FLASH (v1.2.11) [29] was used to merge the sequences. Following that, the high-quality sequences were de-noised using the DADA2 [30] plugin in the Qiime2 [31] pipeline (version 2020.2) to obtain single nucleotide resolution. An amplicon sequence variant (ASV) is a denoised sequence of DADA2. The SILVA 16S rRNA database (v138) was used to classify ASVs using the Naive Bayes consensus taxonomy classifier in Qiime2. Microbial diversity was examined using rarefaction curves and alpha diversity indices (observed ASVs, Chao1, Shannon index, and Good’s coverage) calculated with Mothur v1.30.2 [32]. Based on Bray-Curtis dissimilarity, principal coordinate analysis (PCoA) was used to assess the similarity between microbial communities.

### Untargeted LC–MS-based metabolomics and data preprocessing

The LC-MS/MS metabolite profiling was performed using a Thermo UHPLC-Q Exactive HF-X system with an ACQUITY HSS T3 column (100 mm × 2.1 mm i.d., 1.8 μm, Waters, USA) at Majorbio Bio-Pharm Technology Co. Ltd. (Shanghai, China). Initially, 100 μL of the sample was combined with 400 μL of a 1:1 (v/v) acetonitrile:methanol solution containing 0.02 mg/mL L-2-chlorophenylalanine as an internal standard in a 1.5 mL centrifuge tube for metabolite extraction. Vortex mixing for 30 s and sonication at low temperature for 30 min (5°C, 40 KHz) were applied. After freezing at −20°C for 30 min to precipitate proteins, the samples were centrifuged at 13,000 g and 4°C for 15 min. The clear supernatant was dried under nitrogen, reconstituted in 100 µL of acetonitrile: water (1:1), sonicated again at 5°C for 5 min, and centrifuged to clarify. The final supernatant was transferred to analysis vials. A pooled QC sample, combining equal aliquots from each sample, was analyzed alongside the experimental samples for system conditioning and quality control.

Progenesis QI (Waters Corporation, Milford, USA) software was used to preprocess LC/MS raw data and export a three-dimensional data matrix in CSV format. There were three dimensions in the matrix including sample information, metabolite name and mass spectral response intensity. The internal standard peaks were removed from the data matrix, deredundant, and peak pooled, along with any known false positives (such as noise, column bleed, and derivatized reagents peaks). A database search was also conducted to identify the metabolites, and the main databases were HMDB (http://www.hmdb.ca/), Metlin (https://metlin.scripps.edu/) and Majorbio (https://www.majorbio.com/).

The processed data matrix was uploaded to the Majorbio cloud platform for further analysis. Metabolic features present in at least 80% of any sample group were kept. Values below detection limits were substituted with the minimum detected value, and all features were normalized to the total intensity per sample. To mitigate sample preparation and instrument variability, sum normalization was applied. QC variables with an RSD greater than 30% were removed, and data were log10 transformed. The cleaned data matrix, featuring 810 unique metabolites from both ionization modes, was prepared for in-depth analysis. It is important to note that in our analysis, the differentiation between host-derived and microbial-derived metabolites was not explicitly performed, resulting in a comprehensive profile of both host and microbial metabolites in the metabolomic data.

Using the R package ‘ropls’ (Version 1.6.2), a principal component analysis (PCA) and an orthogonal least partial squares discriminant analysis (OPLS-DA) were performed. A 7-cycle interactive validation assessed the model’s validity. The metabolites with importance in the projection of >1, fold change >1.2 or <0.833, and *P*_*fdr*_ <0.05 were determined as significantly different metabolites based on the VIP obtained by the OPLS-DA model and the *P*-value generated by Wilcoxon rank-sum test and were corrected for multiple comparisons using the Benjamini–Hochberg method.

### Statistical analysis

Statistical evaluations were conducted in R version 4.2.3. Continuous data were analyzed using the two-tailed Wilcoxon rank-sum test, while categorical data were examined with either chi-square or Fisher’s exact test, depending on the data distribution. The Vegan package (v2.4.3) facilitated the execution of the permutational multivariate analysis of variance (PERMANOVA) test to calculate the variation percentage attributable to the treatment and its significance. Discriminatory bacterial taxa between groups were pinpointed through the linear discriminant analysis effect size (LEfSe) method [33] (http://huttenhower.sph.harvard.edu/LEfSe) with linear discriminant analysis (LDA) score >2.5, *p* < 0.05. The MaAsLin2 (Multivariate Association with Linear Models 2) approach was utilized to control for age and gender influences in the taxonomic assessment, using the Linear Model (LM) as the analysis method and default parameters. Functional predictions of the metagenome were made using PICRUSt2 (Phylogenetic Investigation of Communities by Reconstruction of Unobserved States), leveraging ASV representative sequences [34] This process involves multiple steps: aligning ASV sequences to reference databases via HMMER, placing ASVs into a phylogenetic tree with EPA-NG and Gappa, normalizing for 16S rRNA gene copy numbers using castor, and inferring gene family abundances and pathway contributions with MinPath, all in accordance with PICRUSt2 guidelines. The analysis outcomes were analyzed and visualized using the STAMP software (version 2.1.3), applying Welch’s t-test for comparing two groups. Adjustments for multiple comparisons were made using the Benjamini–Hochberg method (*P*_*fdr*_ < 0.05) to ensure statistical rigor.

Identified differential metabolites were integrated into biochemical pathways through metabolic enrichment and pathway analysis, utilizing the KEGG database (http://www.genome.jp/kegg/). These metabolites were categorized based on their associated pathways or functions. Enrichment analysis evaluated whether a set of metabolites collectively participates in specific functional nodes, transitioning from the annotation of individual metabolites to the collective annotation. The enrichment analysis, aimed at identifying biologically relevant pathways influenced by experimental treatments, was conducted using the ‘scipy.stats’ module from Python packages, available at the scipy documentation website.

A procrustes analysis was executed on the microbiota and metabolite data pairs using the Vegan package (v2.4.3). Partial Spearman correlation analysis was utilized to explore the associations between microbiota and metabolites, correcting for the influences of gender and age.

For identifying central microbes/metabolites in OLP, we employed four distinct machine learning methods: least absolute shrinkage and selection operator (LASSO) regression, support vector machine recursive feature elimination (SVM-RFE), extreme gradient boosting (XGBoost), and random forest (RF). LASSO regression was conducted by the R package ‘glmnt’, generating diagnostic biomarkers candidates with tenfold cross-validation [35]. With the R package ‘e1071’, SVM-RFE was conducted. The diagnostic biomarker candidates with the least tenfold cross-validation errors were selected. R package ‘XGBoost’ was used to conduct XGBoost, and top 20 biomarkers were chosen as diagnostic biomarkers candidates. A post-hoc SHAP analysis was performed for interpretation [36]. Random forest was conducted by the R package ‘randomForest’ [37]. The consensus biomarkers identified across these methods were highlighted as key biomarkers and depicted using a flower plot. An artificial neural network (ANN) model was developed using these biomarkers with the ‘neuralnet’ R package [38,39]. The ANN model was designed with three input layers, three hidden layers, and two output layers. This model’s efficacy was assessed on a test set, including receiver operating characteristic (ROC) curve analysis and the area under the curve (AUC) evaluation to measure biomarker performance. Biomarker abundances underwent centered log-ratio (CLR) transformation, with zero values adjusted to 1e-05.

## Results

### Clinical characteristics of recruited subjects

**Figure 1.**
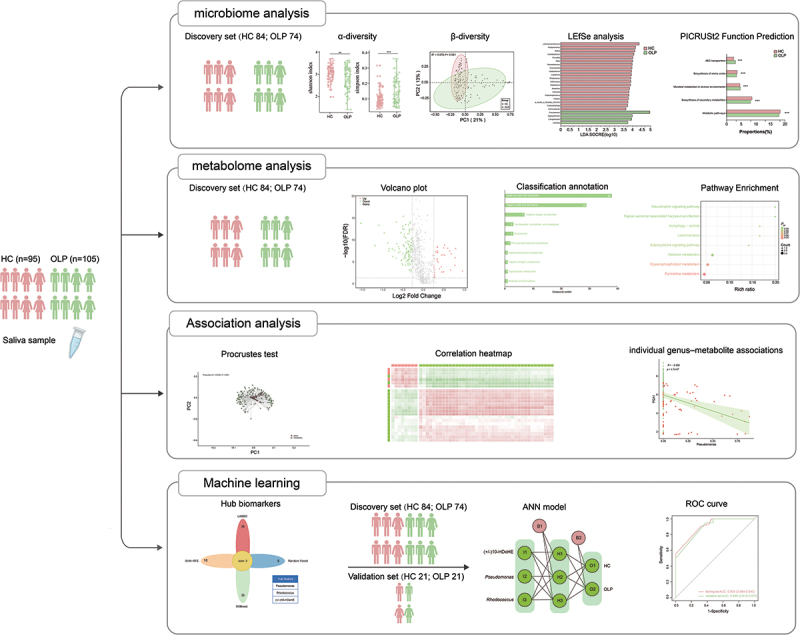
The workflow diagram for this work. Abbreviation: OLP, oral lichen planus; HC, healthy control; LEfSe, linear discriminant analysis effect size; ANN, artificial neural networks; ROC, receiver operating characteristic.

In this study, we analyzed saliva samples from 200 individuals using 16S rRNA gene amplicon sequencing and untargeted metabolomic profiling (Figure 1). These samples were randomly split into a discovery set (158 samples: 74 OLP patients, 84 healthy controls) and a validation set (42 samples: 21 OLP patients, 21 healthy controls). Table 1 presents detailed demographic and clinical features of the participants.

**Table 1.** Characteristics of study cohort.

Variables	Discovery set			Test set			
	HC	OLP	*P*^a^	HC	OLP	*P*^a^	*P*^b^
Samples	84	74	-	21	21	–	
Gender (male %)*	36 (54.5%)	30 (45.5%)	0.768	11 (52.4%)	10 (47.6%)	0.758	–
Age, years^†^	46.14 ± 13.06	45.74 ± 11.47	0.856	44.71 ± 11.30	45.10 ± 11.35	0.95	–
Reticulated lesions^†^	–	3.97 ± 2.39	–	–	3.86 ± 2.67	–	0.666
Hyperemia lesions, cm^2†^	–	0.29 ± 1.03	–	–	0.26 ± 0.68	–	0.464
Erosion lesions, cm^2†^	–	0.53 ± 3.49	–	–	1.48 ± 6.54	–	0.886
Severity scores^†^	–	5.45 ± 7.26	–	–	7.20 ± 13.57	–	0.497

*n (%), †mean ± SD. Continuous, not normally distributed variables between two groups were analysed by Wilcoxon rank-sum test. Categorical variables were compared by the χ^2^ test. - not available. ^a^*p* < 0.05 for equality between OLP vs. HC. ^b^*p* < 0.05 for equality between Discovery set vs. Test set.

### Oral bacteria differences between OLP and HC subjects

**Figure 2.**
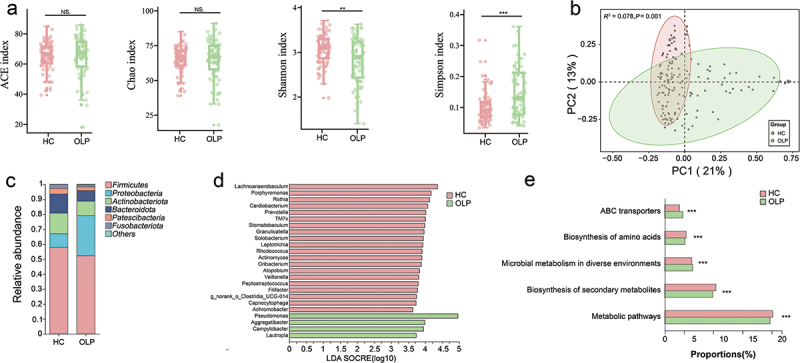
Differential oral microbial characteristics in oral lichen planus (OLP) and health controls (HC). (a) box plots of α-diversity showed that there are significant differences in Shannon and Simpson indices between OLP and HC groups (Wilcoxon rank-sum test). (b) PCoA based on the Bray-Curtis distance at ASV level showed a clear separation between OLP group and HC group (PERMANOVA, *p* = 0.001). (c) the distribution bar plot of the relative abundance of different phyla. (d) using LEfSe analysis, the genera responsible for the discrimination between OLP and HC were identified (LDA > 2.5, *p* < 0.05). (e) the average abundance of the top five KEGG pathways with the highest abundance showed significant differences (Wilcoxon rank-sum test, *P*_*fdr*_ < 0.05). *P_*fdr*_ < 0.05, **P_*fdr*_ < 0.01, ***P_*fdr*_ < 0.001.

To illustrate microbial community richness and species diversity, we used alpha diversity indices, including the Ace index, Chao1 index, Shannon index, and Simpson index, all calculated at the ASV level. The OLP group had a significantly lower Shannon index and a significantly higher Simpson index than the HC group. However, there was no significant difference in the Ace index and Chao1 index between the two groups. These findings imply a decline in alpha diversity among OLP patients (Figure 2a).

To determine if there were distinct microbial differences between OLP and HC, *β*-diversity analysis was performed. Principal Coordinate Analysis (PCoA) revealed a distinct separation in beta diversity of the two groups at the ASV level (Figure 2b, permutational multivariate analysis of variance, *R*^*2*^ = 0.078, *p* = 0.001).

Next, the relative abundance of microbial compositions was compared at the phylum, family and genus levels between the OLP and HC groups in the discovery set. At the phylum level, the OLP group exhibited a significant enrichment of *Proteobacteria* and *Campilobacterota*, while the HC group had higher abundances of *Actinobacteriota*, *Bacteroidota*, *Patescibacteria* and *Fusobacteriota*. The levels of *Firmicutes* remained consistent between both groups (Figure 2c, Supplementary table S1). At the family levels, 18 families displayed differential abundance between the OLP and HC groups. Additionally, at the genus levels, 23 genera showed differential abundance between the OLP and HC groups (Supplementary table S1).

In order to identify the distinct bacterial taxa between the two groups, LEfSe analysis was applied. Based on LDA effect size (LDA >2.5, *p* < 0.05), 24 genera were identified as significantly associated with OLP. Among them, *Pesudomonas*, *Aggregatibacter*, *Campylobacter* and *Lautropia* were significantly enriched in OLP group, while *Lachnoanaerobaculum*, *Porphyromonas*, *Rothia*, *Cardiobacterium*, and *Prevotella*, etc. were mostly related to the healthy individuals. These were consistent with the results obtained from the Wilcoxon test analysis (Figure 2d, Supplementary table S2).

To further validate the results, MaAsLin2 was then applied to control the potential confounding factors, including gender and age. Twenty-two out of 24 genera still exhibited statistically significant associations (*P*_*fdr*_ < 0.05, MaAsLin2, Supplementary table S3). Moreover, the analysis indicated that the genus *Pesudomonas* had the most significantly associated with OLP (Coefficients = 2.77, *P*_*fdr*_ < 0.05). Furthermore, PICRUSt2 was used to predict the functional potential changes for OLP. Among 257 pathways that displayed significant differences between the OLP and Control groups (*P*_*fdr*_ < 0.05), 126 were observed to have markedly elevated activities in the OLP group (Supplementary table S4), such as Two-component system, ABC transporters, Lipopolysaccharide biosynthesis, Bacterial secretion system and Biofilm formation. The top five pathways with the highest abundance are illustrated in Figure 2e. These results suggest that bacteria may play an important role in the pathogenesis of OLP, particularly by modulating pathways related to competition for nutrients, intracellular survival, and replication.

### Oral metabolites alterations in OLP patients

**Figure 3.**
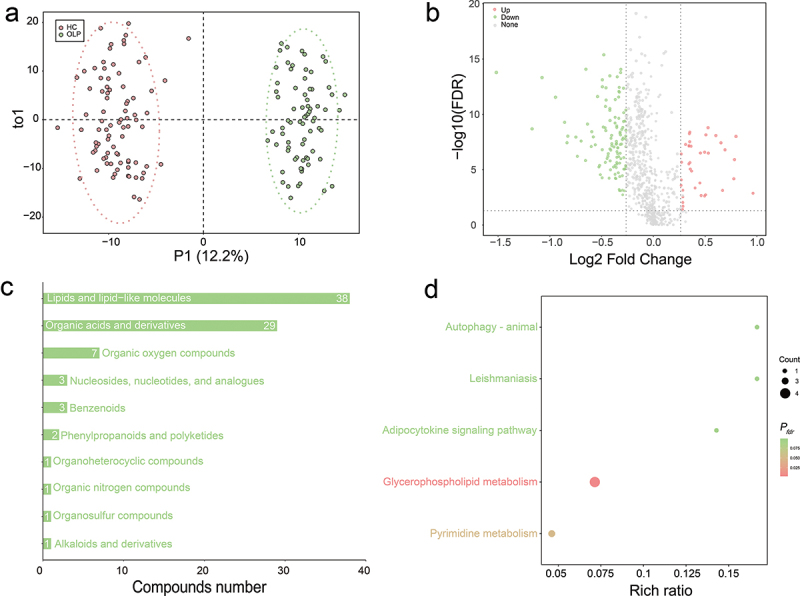
Saliva metabolome changes in oral lichen planus (OLP) and health controls (HC). (a) OPLS-DA of OLP and HC groups. (b) volcano plot demonstrated metabolites changes in OLP compared with HC. Log2-transformed fold change in saliva metabolite abundance is shown on the X axis, and *P*_*fdr*_ analysed using Wilcoxon rank-sum test are shown on the Y axis. Red and green highlights indicate elevated or decreased metabolites. The horizontal lines represent *P*_*fdr*_ < 0.05 and vertical lines indicate fold change of > 1.2 or <0.833. (c) classification annotation of metabolites in HMDB database. (d) KEGG metabolic pathway enrichment analysis based on overlapping metabolites. Points were colored according to adjusted *p* values and sized according to the enriched metabolites in pathways. The X axis indicates the ratio of the number of differential metabolites in a specific pathway to the total number of metabolites in that pathway. *p* values were adjusted using the Benjamini-Hochberg (BH) method. *P*_*fdr*_ < 0.1 was considered statistically significant.

To further refine the characteristics of OLP, we carried out untargeted metabolomics profiling of saliva samples and identified 810 compounds likely from both the microbiome and host, as well as some from ingest compounds. According to PCA score plot (Supplementary figure S1), all QC samples clustered closely after Pareto scaling of the ion feature, demonstrating the excellent repeatability of the analysis. OPLS-DA was used to assess the differences between groups in global metabolic profiles. The scores plot revealed a significant separation of oral metabolites between OLP and HC groups (Figure 3a, Supplementary figure S2). Compared to HC, 153 metabolites showed significant differences in the OLP group, with 38 being up-regulated and 115 down-regulated (*P*_*fdr*_ < 0.05, FC > 1.2 or FC < 0.833, Figure 3b, Supplementary table S5). These metabolites are mostly lipids and lipid-like molecules and organic acids and derivatives (Figure 3c, Supplementary table S5).

To investigate the metabolic pathways involved in the onset and progression of OLP, the 153 differential metabolites were used for KEGG pathway enrichment analysis. The results indicated significant perturbations in several pathways in the OLP group compared to the HC group, including lipid metabolism, nucleotide metabolism, endocrine system, transport and catabolism, and infectious diseases pathways (*P*_*fdr*_ <0.1, Figure 3d, Supplementary table S6). Specifically, in the glycerophospholipid metabolism pathway, we observed distinct alterations: Phosphatidyl-ethanolamine and Phosphatidyl-glycerol were downregulated, while Phosphatidyl-glycerophosphate and CDP-diacyl-glycerol experienced an upregulation. Phosphatidyl-ethanolamine was also found to be downregulated in the Autophagy – animal pathway. In pyrimidine metabolism, metabolites Pseudouridine, Thymidine, and 5-Methyluracil were similarly downregulated in the OLP group. Furthermore, a significant downregulation of N-Palmitoylsphingosine was detected in the OLP group, which is enriched in both the Adipocytokine signaling and Leishmaniasis pathways. Additionally, it was found that three of the five pathways significantly enriched in the metabolome analysis overlapped with the PICRUSt2 predictions: Adipocytokine signaling pathway, Pyrimidine metabolism, and Glycerophospholipid metabolism. This overlap provides a more robust validation of the results.

### Associations of salivary metabolic profiles with the microbiota

**Figure 4.**
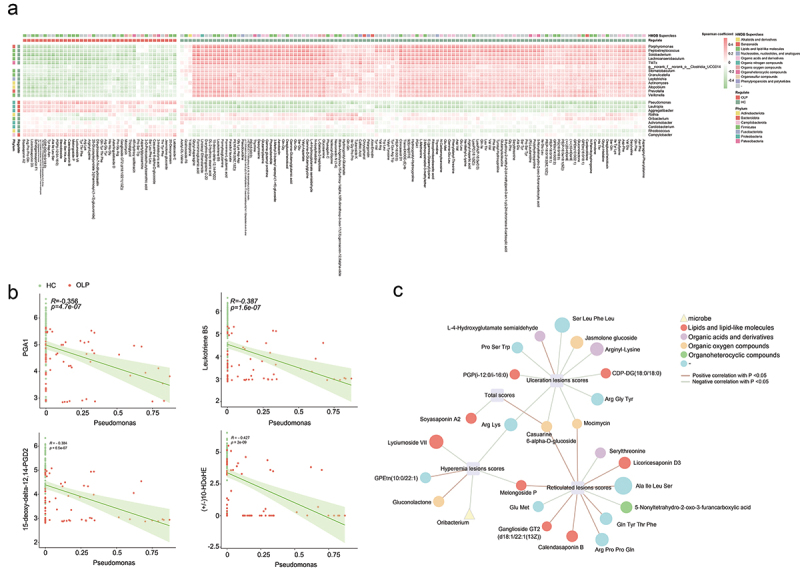
Associations among differentially abundant microbes, differentially metabolites and HRU scale. (a) the heatmap depicts relationship between the differentially abundant microbes and differentially metabolites (partial Spearman analysis). (b) Example of individual genus-metabolite associations. Each dot represents one sample. (c) Network showed significant associations (*p* < 0.05, adjusting for age and gender) between differentially abundant microbes or metabolites and RHU scale. A positive correlation is indicated by a red line, while a negative correlation is indicated by a green line. The circles represent the metabolites, the yellow triangle represent the genus, and purple square represent the RHU scale. The color of circles show the different categories of the metabolites, and the size of circles represents the size of correlation.

As a result of mutual interactions or bidirectional modulation, saliva microbes and metabolites can co-vary [40]. Next, the potential correlations of abundances of these salivary microbes and metabolites were explored. Using Procrustes analysis, a close relationship between salivary microbes and metabolites was found (M^2^ = 0.936, *p* = 0.001, Supplementary figure S3). Subsequently, partial Spearman correlation analysis was performed to examine the association between differentially abundant genera and metabolites. The heatmap showed the association between the differentially abundant genera and metabolites (Figure 4a). In general, out of 153 differential metabolites, strong correlations were observed between 140 metabolites and 18 differential genera (correlation ≥0.3 or ≤ −0.3). These metabolites including several categories: 35 lipids and lipid-like molecules, 29 organic acids and derivatives, 6 organic oxygen compounds, and 5 organoheterocyclic compounds, among others (Supplementary table S7).

Specifically, strong negative associations were found between *Pseudomonas* and (±)10-HDoHE, the latter of which has been reported to be an anti-inflammatory nutrient [41]. Notably, Leukotriene B5, an EPA-derived eicosanoids elevated in control group, was negatively associated with OLP-enriched genera, such as *Pseudomonas* and *Lautropia*. Moreover, overabundance of *Pseudomonas* was negatively correlated with 15-deoxy-delta-12,14-PGD2 and PGA1, which have been shown to display protective effects against inflammation (Figure 4b, Supplementary table S7) [42,43].

### Associations of salivary metabolic profiles and microbiota with RHU scale

Based on partial Spearman correlation analysis, the correlation between disease-associated microbes and metabolites and the RHU scale was investigated (Figure 4c, Supplementary table S8). In reticular lesions scores, Ganglioside GT2 (d18:1/22:1(13Z)), Calendasaponin B, Licoricesaponin D3, Melongoside P, Ala Ile Leu Ser, Gln Tyr Thr Phe, and Arg Pro Pro Gln were positive associated with reticular lesions scores, while Serylthreonine, 5-Nonyltetrahydro-2-oxo-3-furancarboxylic acid, and Glu Met were negatively correlated withreticular lesions scores.

For hyperemic lesions scores, the levels of Lyciumoside VII, Melongoside P, Arg Lys, and the abundance of *Oribacterium* were negatively correlated with hyperemic lesions scores, while the level of Gluconolactone and GPEtn(10:0/22:1) showed a positive correlation. Additionally, Soyasaponin A2 exhibited a negative association with the total scores, and Casuarine 6-alpha-D-glucoside showed a positive association.

For ulceration lesions scores, the higher level of L-4-Hydroxyglutamate semialdehyde companied with increased erosive lesion scores, while the higher levels of Arginyl-Lysine, CDP-DG(18:0/18:0), PGP(i-12:0/i-16:0), Jasmolone glucoside, Casuarine 6-alpha-D-glucoside, Arg Gly Tyr, Arg Lys, Pro Ser Trp, Ser Leu Phe Leu and Mocimycin were associated with decreased erosive lesions scores.

### Combinatorial biomarkers for discriminating OLP from HC

**Figure 5.**
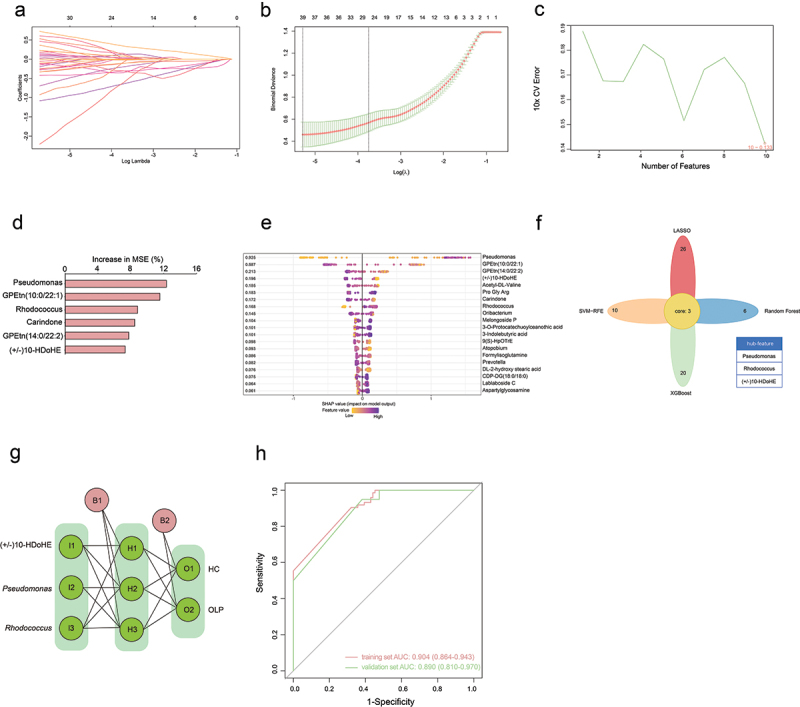
Identification of hub biomarkers in OLP. (a) least absolute shrinkage selection operator (LASSO) coefficient profiles. (b) LASSO regression parameter selection using ten-fold cross-validation. (c) cross-validation error estimation using SVM-RFE tenfold. (d) variable importance plots from random forest models calculated as percentage increases in mean-square errors (MSE). (e) visualizing the importance of features using the XGBoost algorithm with SHAP plot. (f) an intersection diagram of the four above-mentioned machine learning outputs. (g) visualizing artificial neural networks. (h) training and inner validation datasets were verified using receiver operator curves (ROC).

To determine whether the saliva flora and metabolites can be regarded as biomarkers for distinguishing OLP from HC, four machine learning algorithms were utilized to identify the hub microbes and metabolites (Figure 5a–). Using the significantly differential genera and metabolites as inputs for LASSO regression, SVM-RFE algorithm, XGBoost algorithm and random forest algorithm, two genera (*Pseudomonas* and *Rhodococcus*) and one metabolite (±)10-HDoHE were identified by intersecting the outputs of the above four algorithms (Figure 5f). Then, an ANN model based on the features was constructed to discriminate OLP from HC (Figure 5g). The model accuracy was verified using a training set and a validation set. ROC curves were plotted (Figure 5h), and the ANN model demonstrated a high discriminatory ability in the training set, as evidenced by an area under the curve (AUC) value of 0.904, and the validation set (AUC = 0.890). It appears that the combination of hub biomarkers can be viewed as a viable noninvasive diagnostic biomarker due to its robust discriminative performance.

## Discussion

In this study, microbiomics and metabolomics analyses were integrated to investigate alterations in the oral microbiome and metabolome of OLP patients. Distinctive oral microbial compositions and salivary metabolite profiles were identified in OLP patients, highlighting potential biomarkers for diagnosis and treatment. Dysregulated microbes were associated with alterations in metabolites linked to lipid and pyrimidine metabolism, suggesting a potential role in OLP’s inflammatory processes. These findings suggest that oral microbiota disturbances potentially contribute to the inflammatory process of OLP by modulating the host’s lipid metabolism and pyrimidine metabolism, which provides a new insight by which to understanding the pathogenesis of OLP.

The salivary microbiome dysbiosis of OLP was defined by increased relative abundances in *Pseudomonas*, *Aggregatibacter*, *Campylobacter* and *Lautropia*, along with decreased abundances in 18 other genera in this study. *Pseudomonas* is a potential pathogenic taxon associated with systemic inflammation and infection [44]. *Pseudomonas*, a taxon linked to systemic inflammation and infections, produces pyocyanin, a redox-active compound causing cellular harm and immune suppression via reactive oxygen species (ROS) [45,46]. *Aggregatibacter* has been confirmed to be involved in the inflammatory response to periodontitis by stimulating macrophages via the production of pro-inflammatory cytokines [47]. Previous studies have reported that *Campylobacter* is a genus of pro-inflammatory bacteria [48]. Some species of *Campylobacter* have been documented to be associated with chronic intestinal inflammation, reactive arthritis, and systemic inflammation [49–52]. The increased mucosal barrier permeability may allow such bacteria to breach damaged mucosa, thus gaining access to the bloodstream [53,54]. Since OLP is an immune-related disease, the elevated colonization of *Lautropia* might be related to the local immune dysfunction of OLP [55]. Anaerobes such as *Actinomyces*, *Lachnoanaerobaculum*, *Prevotella*, and *Veillonella* were observed to deplete in OLP group, which could produce short-chain fatty acids (SFCAs) by carbohydrate hydrolysis or amino acid metabolism [56]. Since SCFAs bear anti-inflammatory qualities, their lowered production in OLP sufferers suggests a reduction in anti-inflammatory responses [57].

OLP-related KEGG metabolic pathways were also identified. A previous study has reported that the overall saliva metabolomic signature of OLP was distinct from that of HC and glycerophospholipid metabolism pathway was associated with the pathogenesis of OLP [22]. Consistent with these findings, our analysis also revealed alterations in glycerophospholipid metabolism, crucial for systemic immune responses and mild inflammatory conditions [58]. Additionally, our study discovered that pyrimidine metabolites, including Pseudouridine, Thymine, and Thymidine, were markedly reduced in OLP patients. Thymine and Thymidine play critical roles in DNA synthesis. A reduction in Thymidine can result in an imbalance of nucleotides, causing DNA damage and leading to apoptosis, a significant aspect of OLP pathology [59,60]. Moreover, RNAs with modified nucleotides such as pseudouridine are recognized for their role in suppressing the innate immune response of the host [61]. The deficiency of pseudouridine may contribute to activation of immune response.

By integrating multi-omics data, our study further revealed a range of host metabolome-microbiome interplays between differentially abundant microorganisms and metabolites involved in lipid metabolism and Pyrimidine metabolism. For example, *Pseudomonas* shows a positive association with glycerophospholipid metabolites like PGP(i-12:0/i-16:0) and CDP-DG(18:0/18:0), while exhibiting a negative association with GPEtn(14:0/22:2) and PG(16:0/18:0). Phosphatidylglycerophosphate (PGP) and Cytidine diphosphate diacylglycerol (CDP-DG) are crucial for Cardiolipin synthesis, which can hinder the inflammatory resolution by opposing an essential anti-inflammatory immune response during infections [62–64]. Phosphatidylethanolamine (GPEtn), an essential multifunctional phospholipid, plays a significant role in autophagy, closely associated with inflammation [65]. Study has reported a decrease in the content of major cytoplasmic membrane phospholipids (phosphatidylcholine and phosphatidylethanolamine) in colonic epithelia in patients with ulcerative colitis. Specifically, the phospholipid phosphatidylglycerol (PG) has demonstrated anti-inflammatory effects in lungs exposed to LPS. *Pseudomonas* is also found to have negative associations with pyrimidine metabolites like Thymine, Thymidine, and Pseudouridine, along with N-Palmitoylsphingosine, a metabolite in the adipocytokine signaling pathway. Adipocytokines, secreted by adipose tissue, act as hormones and cytokines, such as leptin, adiponectin, IL-6, and TNF-alpha, influencing energy balance, inflammation, and immunity [66]. Similarly, other microbes enriched in OLP patients, like *Campylobacter*, *Lautropia*, and *Aggregatibacter*, show comparable metabolic associations. N-Palmitoylsphingosine exhibits positive correlations with *Prevotella* and *Porphyromonas*. Studies suggest that sphingolipids, produced by *Bacteroidetes* members, influence host lipid metabolism [67,68]. Sphingosine could regulate immune cells to protect the tissue from infection [69]. A diminished abundance of these genera in OLP may be associated with decreased sphingosine synthesis.

In analyzing the relationship between microbe/metabolite profiles and the RHU scale, factors such as environmental influences, sex, and age were recognized as crucial in shaping the oral microbiome and its metabolic output. Therefore, we used partial Spearman correlation analysis to control for the influence of gender and age. Results showed that PGP(i-12:0/i-16:0) and CDP-DG(18:0/18:0) were negatively correlated with ulceration lesion scores. PGP and CDP-DG are essential for the synthesis of cardiolipin [70]. It is reported that reactive oxygen species (ROS) generation leads to loss of mitochondrial cardiolipin content [71]. Higher oxidative stress is encountered in OLP-erosive type than OLP-reticular type [72]. We observed a negative correlation between *orbacterium* and hyperemia lesions scores. Research has demonstrated that *orbacterium* was correlated with host immune parameters, such as IL-6, and LPS, a major bacterial component which can trigger a significant immune response [73]. We also found that Soyasaponin A2 was negatively correlated with total scores. Soyasaponin A2, a triterpenoid glycoside commonly found in soybeans, has been shown to reduce inflammation through inhibiting the activation of the PI3K/Akt/NF-kappaB signaling pathway, which is mediated by ROS [74,75].

As well as identifying the bacteria and metabolic profile that characterize OLP, we sought specific microbes and metabolites to differentiate OLP from HC, for further diagnostic development. Toward this end, we identified a signature of 2 genera and 1 metabolite that could distinguish patients with OLP from HCs, with AUC values 0.904 in discovery sets, and 0.890 in test sets by using an integrated machine learning approach. Ensemble methods surpass individual machine learning algorithms by: 1) enhancing the precision and stability of models through the aggregation of multiple model predictions; 2) diminishing overfitting and bolstering the overall model’s generalizability by utilizing the varied strengths of the combined models [76]. In addition, saliva collection has the advantage of being simple, fast, and more acceptable to patients [77]. This saliva-focused approach offers a rapid, noninvasive solution for distinguishing OLP from healthy controls, fulfilling an essential demand for effective diagnostic instruments to facilitate early intervention tactics.

There are several limitations to this study. First, the study is a single-center study, which may limit its generalizability. Second, although gender and age were adjusted for covariates, our results may still be confounded by host lifestyle and physiological characteristics. Future research needs to collect and analyze data on additional potential confounders, including tobacco usage, caries levels, and periodontal disease status, among others. Third, in our analysis of ‘Oral metabolites alterations in OLP patients’, we did not explicitly discriminate between host-derived and microbial-derived metabolites because differentiating them remains a challenge. Future studies integrating metagenomic or metatranscriptomic data with metabolomic profiles could help elucidate the contributions of specific microbial taxa to the observed metabolic changes. Fourthly, since 16S rRNA gene amplification is based on sequencing and analysis of hypervariable regions, functional prediction cannot distinguish strain-specific functionality, which is an important limitation of PICRUSt2 and any amplicon-based analysis. Furthermore, although our data showed potential correlations between the microbiome, metabolome, and clinical parameters, these findings are still preliminary and require further validation. Finally, the resolution of 16S rRNA gene sequencing at the species level is limited. Utilizing metagenomic sequencing could enhance resolution, uncover differences in diversity, and offer deeper insights into the functional capabilities of the microbiome.

## Conclusions

In conclusion, utilizing integrative multi-omics analysis, our study elucidates the changes in the saliva microbiome and metabolites associated with OLP. We identified distinct microbe–metabolite interactions that were underrepresented in previous research. Furthermore, we formulated a predictive model for OLP diagnosis. These findings provide a comprehensive insight into the role of disordered saliva microbiome and metabolome in the development of OLP, and lay the groundwork for further development of a saliva-based microbiota and metabolite clinical diagnostic tool for OLP.

## Supplementary Material

Additional Figure S1.pdf

Additional Figure S3.pdf

Additional Figure S2.pdf

Additional Tables.xlsx

## Data Availability

The 16S rRNA data generated and/or analyzed during the current study are available in the National Center for Biotechnology Information (NCBI) under Bio Project number PRJNA1097766. The metabolomics data have been uploaded to MetaboLights with the accession number MTBLS10699.

## Abbreviations

OLP: oral lichen planus
HC: health controls
OPMDs: oral potentially malignant disorders
OPLS-DA: orthogonal least partial squares discriminant analysis
PERMANOVA: permutational multivariate analysis of variance
BH: Benjamini-Hochberg
PCA: principal component analysis
LC-MS: liquid chromatography-tandem mass spectrometry
LEfSe: linear discriminant analysis effect size
LDA: linear discriminant analysis
MaAsLin: multivariate association with linear models
PICRUSt2: phylogenetic Investigation of communities by reconstruction of unobserved states
LASSO: least absolute shrinkage and selection operator
SVM-RFE: support vector machine recursive feature elimination
XGBoost: extreme gradient boosting
RF: random forest
ANN: artificial neural network
ROC: receiver operating characteristic
AUC: area under the curve
CLR: centered log-ratio
PCoA: principal coordinate analysis
RHU: reticulation, hyperemia and ulceration
PGP: phosphatidylglycerophosphate
CDP-DG: cytidine diphosphate diacylglycerol
GPEtn: phosphatidylethanolamine
SFCAs: short-chain fatty acids
ROS: reactive oxygen species
