# Supplementary material for: Alterations of oral microbiome and metabolic signatures and their interaction in oral lichen planus
Source: J Oral Microbiol. 2024 Oct 30;16(1):2422164. doi: 10.1080/20002297.2024.2422164 (PMC11533246; doi:10.1080/20002297.2024.2422164)
Supplement: Additional Figure S1.pdf [file ZJOM_A_2422164_SM0593.pdf]

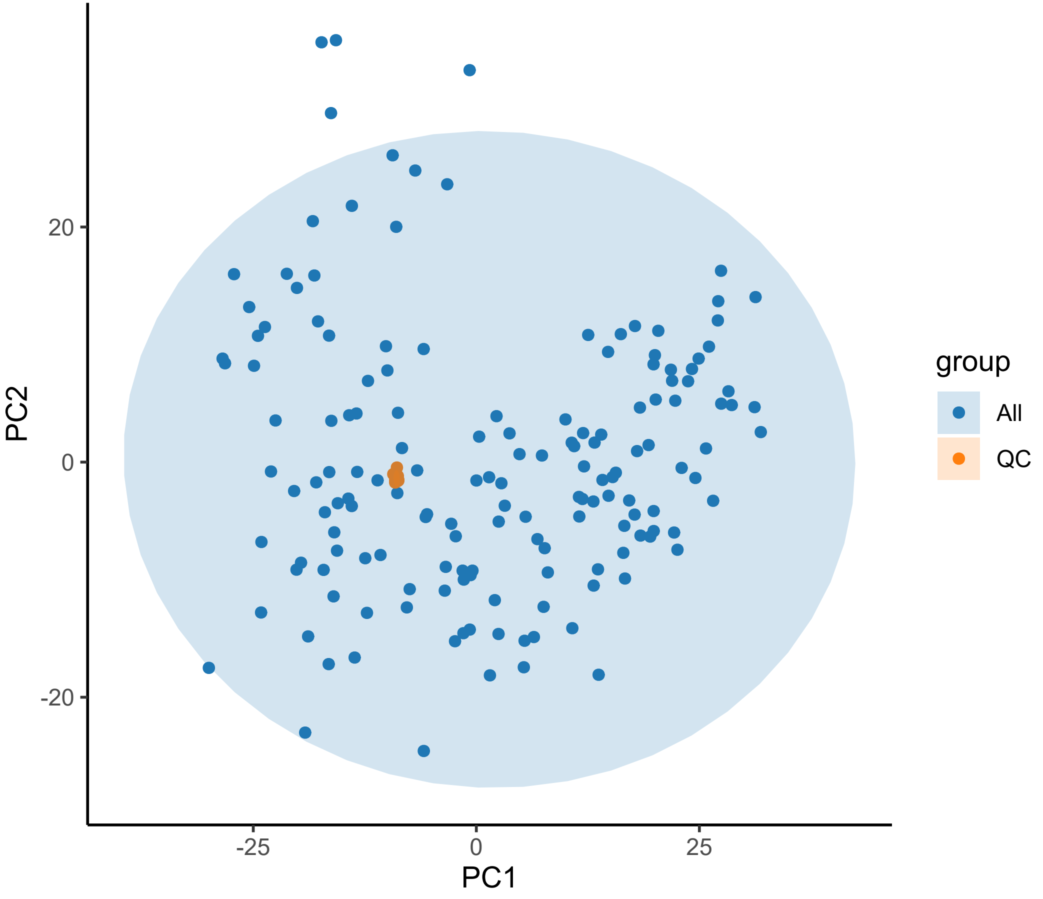

Supplementary Figure S1. PCA of QC samples. The principal component analysis (PCA) on all metabolites revealed consistent metabolic accumulation patterns in quality control (QC) samples, highlighting the reliability of our metabolic profiling.
