# Supplementary material for: Alterations of oral microbiome and metabolic signatures and their interaction in oral lichen planus
Source: J Oral Microbiol. 2024 Oct 30;16(1):2422164. doi: 10.1080/20002297.2024.2422164 (PMC11533246; doi:10.1080/20002297.2024.2422164)
Supplement: Additional Figure S3.pdf [file ZJOM_A_2422164_SM0592.pdf]

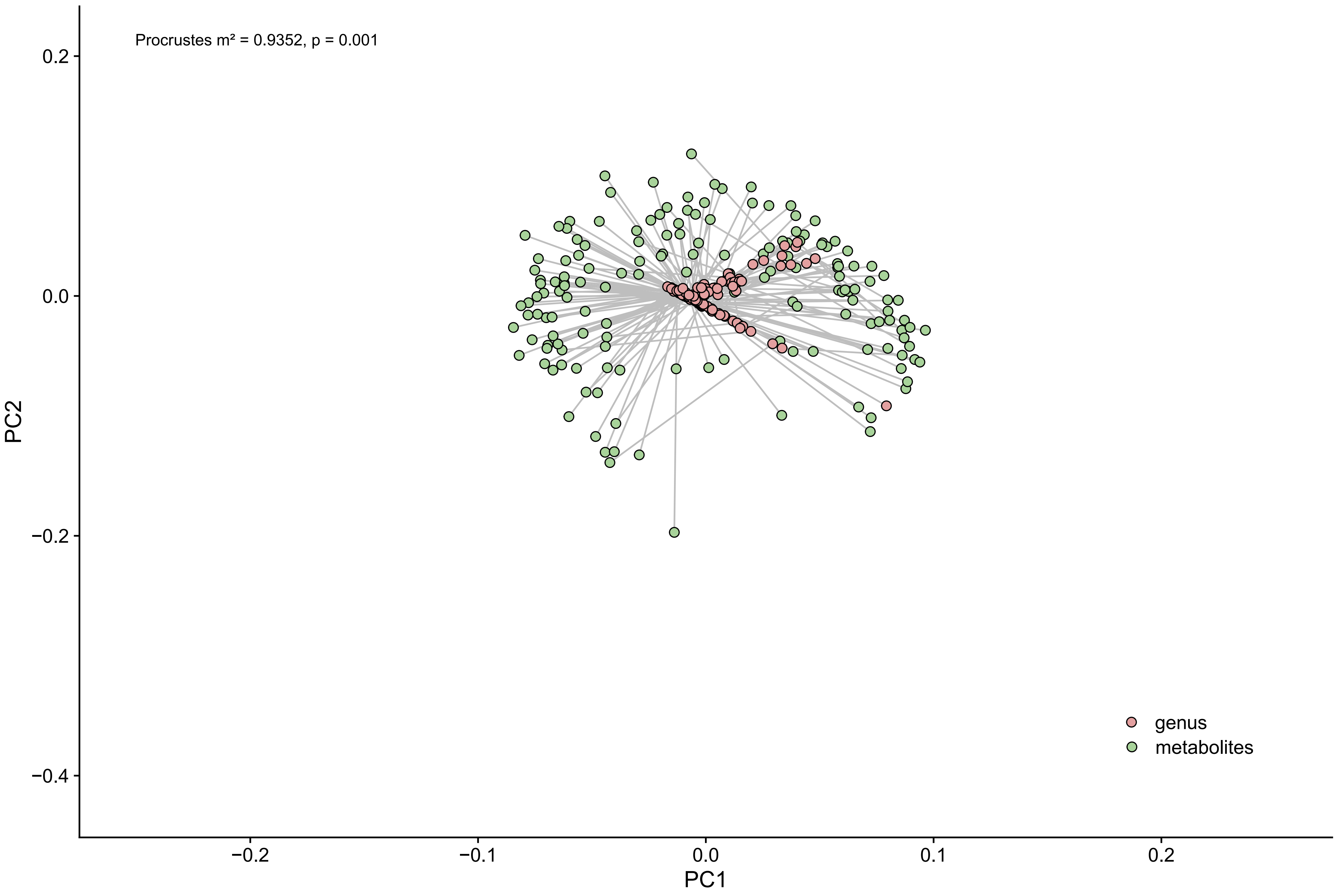

Supplementary Figure S3. Procrustes analyses showing overall association between variation in metabolome and microbiome composition in OLP, with individual samples being connected by line. We used Euclidean distance for metabolome (green circles) and Bray-Curtis distance for microbiome data (red circles), and the procrustes m^2 statistic results were labeled.
