# Supplementary material for: Alterations of oral microbiome and metabolic signatures and their interaction in oral lichen planus
Source: J Oral Microbiol. 2024 Oct 30;16(1):2422164. doi: 10.1080/20002297.2024.2422164 (PMC11533246; doi:10.1080/20002297.2024.2422164)
Supplement: Additional Figure S2.pdf [file ZJOM_A_2422164_SM0591.pdf]

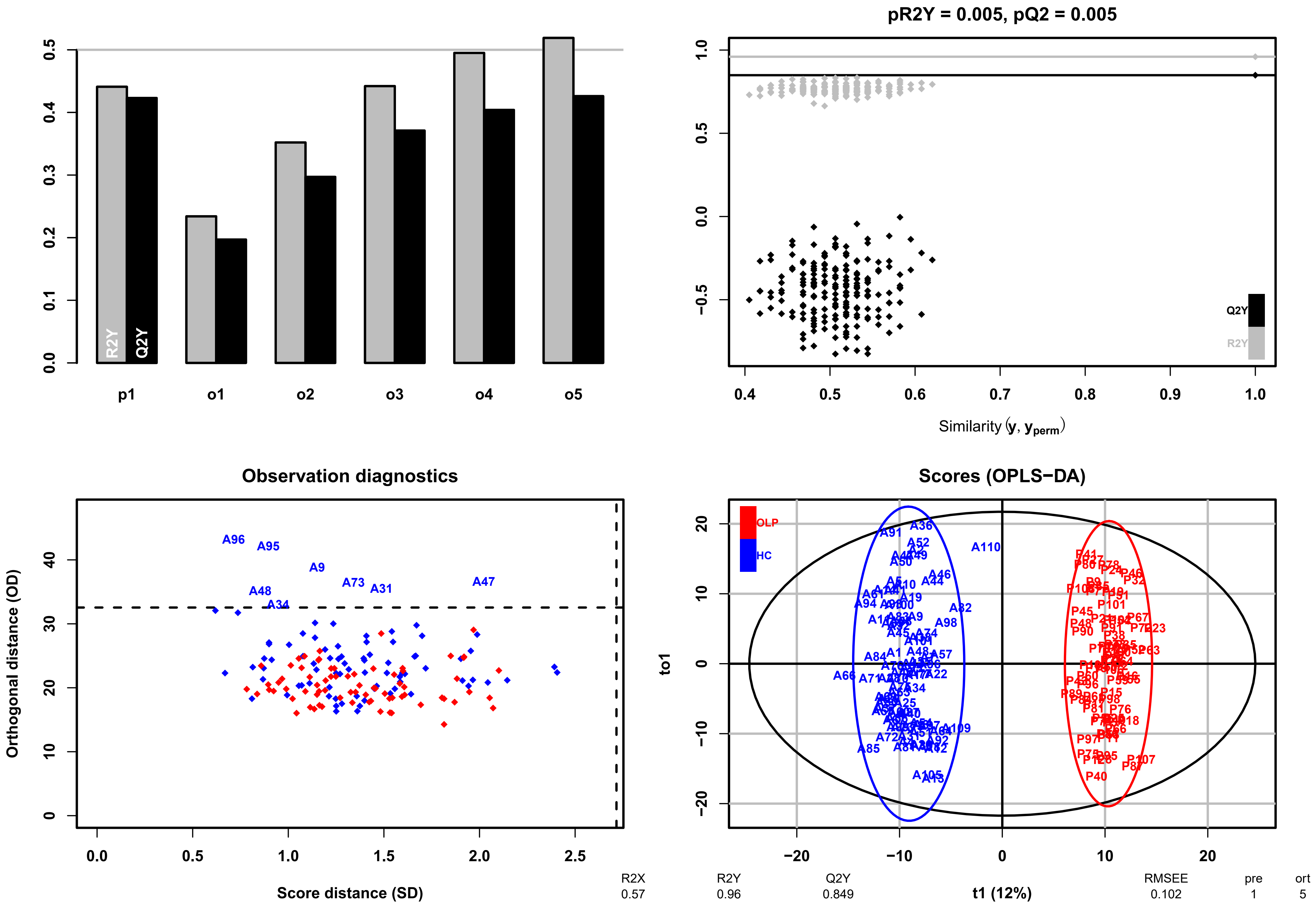

Supplementary Figure S2. OPLS-DA Score Plots and Model Validation Parameters. The OPLS-DA score plot was inspected for model accuracy parameters recording the acceptable goodness of fit/prediction values(i.e. R2X = 0.57; R2Y = 0.66; Q2Y = 0.849).
